# Social Interaction, Life Satisfaction, and Follow-Up Sleep Quality in Community-Dwelling Adults: A Three-Year Longitudinal Cohort Study in Japan

**DOI:** 10.3390/healthcare14152311

**Published:** 2026-07-31

**Authors:** Ruifeng Zhao, Haotian Gao, Shuanghong Li, Yixin Sun, Jinrui Zhang, Maiko Shigeeda, Mengxuan Wang, Ruoyu Zhou, Yuko Sawada, Akihiro Kakuda, Tokie Anme

**Affiliations:** 1 School of Future Education, Qingdao Hengxing University of Science and Technology, Qingdao 266100, China; zhao43218611@gmail.com (R.Z.); zhouruoyu86@gmail.com (R.Z.); 2 Doctoral Program in Medical Sciences, Graduate School of Comprehensive Human Sciences, University of Tsukuba, Tsukuba 305-8577, Japan; s2330446@u.tsukuba.ac.jp (H.G.); s2330440@u.tsukuba.ac.jp (S.L.); sunyixin10@outlook.com (Y.S.); jinrui970308@gmail.com (J.Z.); maiko.shigeeda@gmail.com (M.S.); s2230446@u.tsukuba.ac.jp (M.W.); 3 Department of Physical Therapy, Morinomiya University of Medical Sciences, Osaka 559-8611, Japan; ysawa1110@yahoo.co.jp (Y.S.); kakuda@morinomiya-u.ac.jp (A.K.); 4 Faculty of Medicine, University of Tsukuba, Tsukuba 305-8575, Japan

**Keywords:** sleep quality, sleep restfulness, social interaction, life satisfaction, community-dwelling adults, longitudinal cohort study, nonlinearity, natural cubic spline

## Abstract

**Highlights:**

**What are the main findings?**

- The association between baseline social interaction and three-year follow-up sleep quality was nonlinear, with the strongest association in the lower ISI range and a plateau at higher scores.
- Life satisfaction showed an exploratory indirect association, while evidence of an ISI × life satisfaction effect modification was sensitive to the functional form used for ISI.

**What are the implications of the main findings?**

- Low ISI scores may be more informative for sleep-health research than small score differences near the upper end of the scale.
- The findings are associative and model-dependent and do not establish causal mediation, intervention effects, or predictive screening performance.

**Abstract:**

**Background/Objectives**: Social interaction may be associated with sleep health, but longitudinal evidence among working-age adults is limited, and ceiling-concentrated scores may complicate linear modeling. We examined baseline social interaction, three-year follow-up sleep quality, and the exploratory role of life satisfaction. **Methods**: Data came from 487 adults aged 20–58 years surveyed in 2020 and 2023. Social interaction was assessed using the 18-item Index of Social Interaction (ISI). Normal sleep required at least 6 h of sleep and adequate restfulness. Models were adjusted for baseline sleep, demographics, lifestyle, health, depression, and anxiety. The ISI functional form was assessed using natural cubic splines and a piecewise model with a knot at 14. **Results**: In the initial linear summary model, a higher ISI score was associated with normal follow-up sleep (OR = 1.24, 95% CI: 1.09–1.42). The spline model fit better (likelihood-ratio χ^2^ = 19.69, df = 2, *p* < 0.001). Below 14, each 1-point increase was associated with higher odds of normal sleep (OR = 2.81, 95% CI: 1.64–4.82); no additional association was observed at 14 or higher (OR = 0.99, 95% CI: 0.83–1.19). The ISI × life satisfaction interaction was sensitive to parameterization. Spline-based analysis showed an average indirect association through life satisfaction (estimate = 0.0077, 95% bootstrap CI: 0.0019–0.0153), while direct and total associations were not significant. ISI was associated with restfulness but not duration. **Conclusions**: The ISI–sleep association was nonlinear, concentrated in lower scores, and plateaued near the upper end. Life-satisfaction findings were exploratory; concurrent measurement precluded causal mediation inference.

## 1. Introduction

Sleep quality is an important component of health, well-being, and daily functioning among adults. Poor sleep is associated with emotional distress, impaired daytime functioning, reduced well-being, and poorer quality of life [1,2]. Sleep disturbances are also closely related to symptoms of depression and anxiety, and these relationships may be bidirectional [3]. From a community health perspective, sleep should therefore be considered not only as a physiological or behavioral outcome but also in relation to interpersonal relationships, subjective well-being, and broader psychosocial resources.

In Japan, sleep health has increasingly been emphasized as a public health and health-promotion priority. The Japanese Ministry of Health, Labour and Welfare recommends considering both sleep duration and whether individuals feel adequately rested through sleep [4]. A sleep duration of at least 6 h is an important adult sleep-health criterion, while insufficient sleep restfulness is also clinically and epidemiologically relevant [5,6]. Combining these brief indicators may provide a practical community-based measure of sleep health, although it does not replace a comprehensive sleep instrument or clinical assessment.

Social interaction is a potentially modifiable psychosocial characteristic that may be associated with sleep quality. It includes contact with family, friends, neighbors, and community groups, as well as broader dimensions of participation, social curiosity, independence, and perceived safety [7]. Systematic reviews have linked social relationships, social isolation, and loneliness with sleep outcomes and broader mental and physical health in healthy and older populations [8,9,10]. Japanese longitudinal evidence among older adults has also suggested that stronger social relationships, particularly when combined with physical activity, are associated with a lower risk of subsequent sleep disorders [11]. These findings support examining social interaction as a multidimensional community resource rather than only as the frequency of interpersonal contact.

The meaning and health implications of social interaction may differ across cultural and religious contexts. Religious involvement can combine social participation, shared routines, meaning, emotional support, and stress regulation and has been proposed as a social determinant of sleep [12]. Qualitative evidence from religious Muslim and Jewish adults further illustrates that prayer timing, religious prescriptions, and faith-related stress regulation may influence sleep timing, duration, and perceived quality [13]. Preliminary research in Greek older adults has also examined sleep quality, general health, and religiousness together [14]. During the COVID-19 pandemic, culturally embedded community practices such as rural Japanese Osekkai were associated with maintaining social participation [15]. Likewise, research among older adults has emphasized that life satisfaction reflects physical, social, psychological, and environmental resources whose relative importance may vary across populations [16]. These cross-cultural considerations are relevant when interpreting evidence from Japan; however, religiosity was not measured in the present cohort and could not be examined as a confounder or effect modifier.

Life satisfaction refers to an individual’s cognitive evaluation of overall life quality and is a central component of subjective well-being [17]. Greater social interaction may be associated with stronger perceived support, belonging, and opportunities for meaningful participation, which in turn may correspond to greater life satisfaction [18]. Life satisfaction is also associated with health and longitudinal sleep outcomes [19,20]. Nevertheless, when social interaction and life satisfaction are assessed at the same time, an indirect statistical association cannot establish that social interaction temporally precedes or causes changes in life satisfaction.

Several gaps remain. Much of the available evidence concerns older or clinical populations, while longitudinal evidence among community-dwelling working-age adults is limited. Many studies are cross-sectional, and few have tested whether the association between social interaction and later sleep differs according to life satisfaction. In addition, composite sleep outcomes may obscure whether an association is driven by sleep duration or perceived sleep restfulness. Addressing these issues may clarify how interpersonal and subjective well-being characteristics are associated with later sleep health without assuming causal mediation or predictive performance.

### 1.1. Study Rationale and Healthcare Relevance

This study was guided by a community health framework linking interpersonal resources, subjective well-being, and health-related outcomes. Social interaction was selected as the primary exposure because the ISI captures multiple aspects of everyday social functioning in Japanese community settings, including independence, curiosity, interpersonal contact, participation, and safety [7,21,22]. These dimensions may be associated with emotional support, belonging, meaningful activity, and stable daily routines.

Life satisfaction was examined as a proposed explanatory variable because it provides a brief appraisal of subjective well-being and may statistically account for part of the association between social interaction and subsequent sleep. It was also considered a potential effect modifier because the relevance of additional social resources may differ between adults who are and are not satisfied with their current lives. The study therefore evaluated both an exploratory indirect association and the ISI × life satisfaction interaction.

Follow-up sleep quality was selected as the health outcome because sleep is closely connected with mental health, daily functioning, and quality of life [1,2,3,4,5,6,23]. The study also examined sleep duration and sleep restfulness separately, allowing an assessment of whether psychosocial associations were more evident for behavioral sleep quantity or perceived restoration. This distinction is relevant to community health because the two components may have different correlates and implications.

### 1.2. Aim and Analytical Questions

The aim of this three-year longitudinal study was to examine the association between baseline social interaction and follow-up sleep quality among community-dwelling adults in Japan and to assess the statistical role of baseline life satisfaction.

We examined three primary associations: (1) baseline social interaction with normal sleep quality at follow-up; (2) baseline social interaction with life satisfaction; and (3) life satisfaction with normal sleep quality at follow-up.

Exploratory analyses examined whether life satisfaction statistically accounted for part of the association between social interaction and follow-up sleep quality, whether life satisfaction modified this association, whether the findings differed for sleep duration and sleep restfulness, and whether a linear ISI term adequately represented the association. Because the analyses were not preregistered, the interaction, indirect-effect, and alternative functional-form analyses were interpreted as theoretically informed exploratory analyses rather than confirmatory causal tests.

## 2. Materials and Methods

### 2.1. Study Design and Setting

This study used a three-year longitudinal cohort design and was reported in accordance with the Strengthening the Reporting of Observational Studies in Epidemiology (STROBE) statement. Data were obtained from the Community Empowerment and Care for Well-being and Healthy Longevity cohort study, an ongoing community-based cohort conducted in a suburban area of Japan. The cohort collects self-reported questionnaire data from community residents at three-year intervals. The baseline survey used in the present analysis was conducted in August 2020, and the follow-up survey was conducted in 2023.

### 2.2. Participants and Recruitment

The target population comprised community-dwelling adults aged 20–64 years who participated in the 2020 baseline survey. The final analytic sample ranged from 20 to 58 years of age. Participants were eligible for the present analysis if they completed both the 2020 baseline survey and the 2023 follow-up survey and had available data on social interaction, life satisfaction, sleep quality, and the covariates included in the revised models.

Community residents were identified through the local government’s resident health-survey roster. Local government staff distributed the questionnaires to eligible residents, and volunteers conducted home-visit surveys for residents with mobility limitations. The total number of eligible residents initially invited was not available in the secondary dataset; therefore, the baseline response rate could not be calculated. A total of 796 adults completed the August 2020 baseline survey.

**Figure 1:**
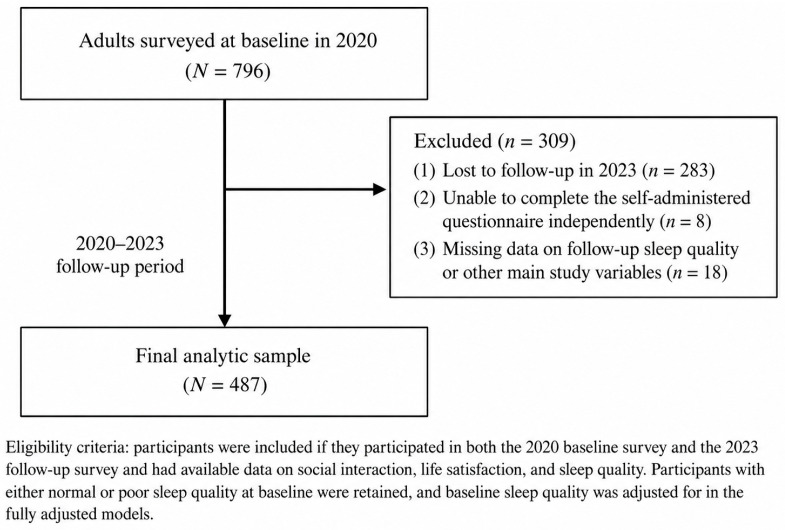
Flow chart of participant selection.

Of the 796 adults surveyed at baseline, 283 did not participate in the 2023 follow-up, eight were unable to complete the self-administered questionnaire independently, and 18 had missing data on the principal study variables. Specific reasons for nonparticipation among the 283 participants lost to follow-up were not available in the secondary dataset. Participants with either normal or poor sleep quality at baseline were retained, and baseline sleep quality was included as a covariate in the primary models. The final complete-case analytic sample comprised 487 participants (Figure 1).

### 2.3. Sampling Approach, Study Size, and Attrition Assessment

This study was a secondary analysis of an existing community-based cohort and did not use probability sampling. No a priori sample-size calculation was conducted because the analytic sample was determined by follow-up participation and complete data availability. The analytic retention rate was 61.2% (487/796). To assess potential attrition and complete-case selection bias, baseline characteristics were compared between participants included in the final analysis and those who were not included. Absolute standardized mean differences were reported alongside unadjusted *p*-values, with larger standardized differences interpreted as indicating greater imbalance.

### 2.4. Measures

#### 2.4.1. Sleep Quality

Sleep duration and sleep restfulness were assessed at baseline in 2020 and at follow-up in 2023. Sleep duration was assessed using the item “Approximately how many hours and minutes do you sleep per day?” Responses were recorded in hours and minutes. Based on the Japanese Ministry of Health, Labour and Welfare sleep health guideline, a sleep duration of at least 6 h was coded as adequate (1), whereas a duration of less than 6 h was coded as short (0) [4]. Sleep restfulness was assessed using the item “Do you think you obtain sufficient rest through sleep?” and was coded as 1 = yes and 0 = no.

Participants were classified as having normal sleep quality when they reported both a sleep duration of at least 6 h and adequate sleep restfulness. Participants who failed to meet one or both criteria were classified as having poor sleep quality. The composite sleep-quality variable was coded as 1 = normal and 0 = poor. Follow-up sleep quality in 2023 was the primary outcome, and baseline composite sleep quality was included as a covariate in the primary analysis.

To examine whether the composite result was driven by one component, adequate sleep duration and adequate sleep restfulness at follow-up were analyzed separately in sensitivity analyses, with adjustment for the corresponding baseline component. Four directional sleep-quality transitions were also described: normal-to-normal, normal-to-poor, poor-to-normal, and poor-to-poor. The previously defined favorable/unfavorable trajectory variable was not retained because it was effectively equivalent to follow-up sleep status.

#### 2.4.2. Social Interaction

Social interaction was assessed at baseline using the Index of Social Interaction (ISI; Supplementary Table S11), a measure developed for community-dwelling populations in Japan [21]. The ISI comprises 18 binary items covering independence in daily life, social curiosity, interaction with others, participation in society, and feelings of safety. Item scores were summed to produce a total score ranging from 0 to 18, with higher scores indicating greater social interaction. The total score was treated as a continuous exposure variable.

The ISI was selected because it was developed for Japanese community settings, captures multiple dimensions of everyday social functioning rather than only network size or frequency of contact, and was already embedded in the longitudinal cohort protocol. Previous research has reported a five-domain structure, acceptable internal consistency (Cronbach’s alpha approximately 0.78), and predictive associations with later functional outcomes [22].

#### 2.4.3. Life Satisfaction

Life satisfaction was assessed at baseline using the item “Are you satisfied with your current life?” Responses were coded as 1 = yes and 0 = no. The item was treated as a brief indicator of subjective well-being rather than a comprehensive psychometric scale. It was examined as a proposed explanatory variable in the exploratory indirect-effect analysis. Because social interaction and life satisfaction were measured at the same baseline wave, temporal ordering between them could not be established.

#### 2.4.4. Measurement Considerations

The measures were selected within the constraints of an ongoing community cohort. The guideline-based sleep indicators were suitable for brief community health assessment and reflected nationally relevant criteria, but they did not provide the symptom detail available from multi-item instruments such as the Pittsburgh Sleep Quality Index or objective assessments such as actigraphy. The ISI was selected for its cultural and community relevance and multidimensional coverage, although its upper score concentration required explicit assessment of the linearity assumption. The single-item life-satisfaction measure minimized respondent burden in a large longitudinal survey but did not capture the full breadth of subjective well-being.

#### 2.4.5. Covariates

Covariates were selected based on prior evidence, theoretical relevance, and availability in the cohort dataset. The adjusted models included baseline age, sex, body mass index (BMI), baseline sleep quality, breakfast consumption, snack intake, salt intake control, attention to nutritional balance, avoidance of high-fat foods, exercise, current smoking, current drinking, hospitalization or prolonged medical treatment during the previous year, depressive symptoms, and anxiety symptoms.

Height and weight were measured at a health examination center operated by the local government, and BMI was calculated as weight in kilograms divided by height in meters squared. Breakfast consumption, snack intake, salt intake control, attention to nutritional balance, and avoidance of high-fat foods were each assessed using a binary yes/no item and coded as 1 = yes and 0 = no. Exercise was coded as 1 for exercising almost every day, three to four times per week, or one to two times per week, and as 0 for almost never exercising. Current smoking was coded as 1 for smoking almost every day or sometimes and as 0 for former or never smoking. Current drinking was coded as 1 for drinking almost every day or sometimes and as 0 for never drinking. Hospitalization/prolonged treatment was coded as 1 when participants reported hospitalization or continuous treatment for at least two weeks during the previous year and as 0 otherwise.

Depressive symptoms were assessed using a binary item asking whether participants had felt down or depressed during the previous year. Anxiety symptoms were assessed using a binary item asking whether participants had experienced anxiety or worry during the previous year. Both were coded as 1 = yes and 0 = no and were treated as brief self-reported symptom indicators rather than clinical diagnoses. The exact assessment items, response options, and coding rules are provided in Supplementary Table S9.

### 2.5. Rationale for Statistical Methods

Logistic regression was selected because follow-up sleep quality and life satisfaction were binary variables. Nonlinear models were used to assess the functional form of the ISI–sleep association, whereas the linear model was retained only as an overall summary of the average association across the observed score range. The outcome model included ISI, life satisfaction, and their interaction because exposure–mediator interaction can alter the interpretation and estimation of direct and indirect associations.

Logistic regression-based natural-effect estimation was used to quantify exploratory direct and indirect associations on the risk-difference scale. The mediator model used a linear ISI term because no evidence of nonlinearity was observed for life satisfaction. The outcome model used a natural cubic spline for ISI and included interactions between the spline terms and life satisfaction. The analysis was intended to assess a local 1-point contrast near the sample mean and was not intended to establish a causal or temporally ordered mediation process.

### 2.6. Statistical Analysis

Continuous variables were summarized using means and standard deviations or medians and interquartile ranges, as appropriate. Categorical variables were summarized using frequencies and percentages. Baseline characteristics were compared descriptively according to follow-up sleep quality using Welch *t* tests, Mann–Whitney U tests, or chi-square tests. These comparisons were not used to select covariates and were interpreted descriptively; *p*-values were unadjusted for multiple comparisons.

Baseline characteristics of participants included in the final analysis were compared with those of participants who were not included using the same descriptive tests. Absolute standardized mean differences were additionally calculated to assess the magnitude of imbalance independently of sample size.

For the initial linear summary, three adjusted logistic regression models were fitted using a linear ISI term. Model 1 estimated the average per-point association between baseline ISI and normal follow-up sleep quality. Model 2 estimated the association between baseline ISI and life satisfaction. Model 3 estimated normal follow-up sleep quality from mean-centered ISI, life satisfaction, and the linear ISI × life satisfaction interaction. All models adjusted for baseline age, sex, BMI, the relevant baseline sleep measure, the five dietary indicators, exercise, current smoking, current drinking, hospitalization/prolonged treatment, depressive symptoms, and anxiety symptoms. Conditional linear ISI associations were estimated separately for participants who were and were not satisfied with their current lives, and the interaction model was compared with the corresponding model without the interaction term using a likelihood-ratio test.

Exploratory natural indirect and direct associations were estimated for a 1-point increase in ISI from the sample mean (16.11) to 17.11. The mediator model contained a linear ISI term, whereas the outcome model contained a natural cubic spline for ISI and spline × life satisfaction interaction terms. Natural indirect and direct associations were estimated at both exposure levels and averaged. The average natural indirect association, average natural direct association, and total local association were reported on the risk-difference scale. Percentile bootstrap confidence intervals were calculated using 3000 resamples. A sensitivity analysis was repeated without adjustment for baseline sleep quality.

Additional sensitivity analyses examined adequate sleep duration and adequate sleep restfulness as separate outcomes, with adjustment for the corresponding baseline component. Four baseline-to-follow-up sleep transitions were summarized descriptively. The ISI functional form was evaluated by comparing the linear model with quadratic and natural cubic spline models; the spline had three effective degrees of freedom. For interpretability after evidence of nonlinearity, a piecewise linear sensitivity model with a knot at ISI = 14 (the sample 10th percentile) and an analysis restricted to ISI scores of at least 14 were also fitted. Model fit was compared using likelihood-ratio tests and the Akaike information criterion. The robustness of the ISI × life satisfaction interaction was examined under linear, spline, quadratic, piecewise, and upper-range-restricted parameterizations using joint likelihood-ratio tests as appropriate. Multicollinearity was assessed using variance inflation factors after mean-centering ISI; values below 2.5 were considered nonproblematic.

Descriptive statistics and group comparisons were performed using IBM SPSS Statistics version 28.0 (IBM Corp., Armonk, NY, USA). Regression analyses, nonlinear modeling, interaction analyses, natural-effect estimation, bootstrap procedures, and sensitivity analyses were conducted using Python version 3.13.5. Data management was performed using pandas version 2.2.3 and NumPy version 2.3.5. Statistical modeling and related procedures used statsmodels version 0.14.6, SciPy version 1.17.0, and patsy version 1.0.2. Figures were generated using Matplotlib version 3.10.8. All statistical tests were two-sided, with *p*-values < 0.05 considered statistically significant. A fixed random seed was used to ensure reproducibility of the bootstrap analyses.

## 3. Results

### 3.1. Participant Flow and Attrition Analysis

Of the 796 adults surveyed at baseline, 487 (61.2%) were included in the final analysis, and 309 (38.8%) were not included because of loss to follow-up, inability to complete the questionnaire independently, or missing principal study variables. Compared with participants who were not included, those retained in the analytic sample were younger (43.84 ± 9.10 vs. 46.21 ± 7.93 years, *p* < 0.001; absolute standardized mean difference [SMD] = 0.28). No statistically significant differences were observed for BMI, ISI score, sex, life satisfaction, the five dietary indicators, exercise, current smoking, current drinking, hospitalization/prolonged treatment, depressive symptoms, anxiety symptoms, or the baseline sleep indicators (Supplementary Table S1).

### 3.2. Participant Characteristics and Sleep Indicators

The 487 participants had a mean baseline age of 43.84 years (SD = 9.10) and a mean BMI of 22.80 kg/m^2^ (SD = 3.69). The median ISI score was 17 [interquartile range: 15–17], indicating a concentration of scores near the upper end of the scale. Women accounted for 59.1% of the sample. At baseline, 221 participants (45.4%) had normal sleep quality, 367 (75.4%) reported life satisfaction, 43 (8.8%) reported depressive symptoms, and 64 (13.1%) reported anxiety symptoms (Table 1).

At follow-up, 210 participants were classified as having poor sleep quality and 277 as having normal sleep quality. Normal baseline sleep quality, life satisfaction, and exercise were more common among participants with normal follow-up sleep quality. Anxiety symptoms were reported by 16.2% of participants with poor follow-up sleep and 10.8% of those with normal follow-up sleep; the unadjusted descriptive comparison was not statistically significant (*p* = 0.083) (Table 2).

At baseline, 87 participants had a sleep duration below 6 h, and 263 reported insufficient sleep restfulness; 84 met both poor-sleep criteria. At follow-up, 85 participants had a sleep duration below 6 h, and 205 reported insufficient sleep restfulness; 80 met both criteria (Supplementary Table S2). The four directional transitions were normal-to-normal (*n* = 179, 36.8%), normal-to-poor (*n* = 42, 8.6%), poor-to-normal (*n* = 98, 20.1%), and poor-to-poor (*n* = 168, 34.5%) (Supplementary Table S4).

### 3.3. Initial Linear Summary Associations and Exploratory Interaction

In the initial linear summary model, each 1-point increase in baseline ISI was associated with higher odds of normal sleep quality at follow-up (OR = 1.24, 95% CI: 1.09–1.42, *p* < 0.001). This coefficient represents an average per-point association across the observed ISI range rather than a constant association at every score. Higher ISI was also associated with life satisfaction (OR = 1.32, 95% CI: 1.16–1.51, *p* < 0.001). In the linear outcome model containing the interaction, life satisfaction was associated with normal follow-up sleep at the mean ISI score (OR = 4.78, 95% CI: 2.71–8.44, *p* < 0.001).

The linear ISI × life satisfaction interaction was statistically significant (OR = 0.69, 95% CI: 0.50–0.95, *p* = 0.022), and adding the interaction improved model fit (likelihood-ratio χ^2^ = 5.93, *p* = 0.015). In this linear parameterization, the ISI association was stronger among participants who were not satisfied with their current lives (OR = 1.50, 95% CI: 1.14–1.97, *p* = 0.003) than among those who were satisfied (OR = 1.03, 95% CI: 0.87–1.23, *p* = 0.698) (Table 3). Because the interaction was not consistent under all nonlinear parameterizations, these subgroup estimates were interpreted as exploratory. Anxiety symptoms were not independently associated with follow-up sleep quality in the fully adjusted models (Supplementary Table S6).

### 3.4. Spline-Based Exploratory Direct and Indirect Associations

In the spline-based natural-effect analysis, the average indirect association through life satisfaction was statistically significant for a local 1-point increase in ISI from 16.11 to 17.11 (estimate = 0.0077, Boot SE = 0.0034, 95% bootstrap CI: 0.0019–0.0153, *p* = 0.009). The average direct association was not statistically significant (estimate = 0.0115, 95% bootstrap CI: −0.0403–0.0637, *p* = 0.689), and the total local association was also not statistically significant (estimate = 0.0192, 95% bootstrap CI: −0.0336–0.0717, *p* = 0.464) (Table 4). These estimates represent exploratory local statistical associations under a nonlinear outcome model rather than evidence of causal mediation.

### 3.5. Nonlinearity and Sensitivity Analyses

The linearity-in-the-logit assumption was not supported for ISI in the follow-up sleep model. A natural cubic spline model fit better than the single linear term (likelihood-ratio χ^2^ = 19.69, df = 2, *p* < 0.001; AIC = 557.01 vs. 572.70). Adjusted normal-sleep probability rose sharply across the lower ISI range and then plateaued (Supplementary Figure S1). In the piecewise model with a knot at ISI = 14, each 1-point increase below 14 was associated with higher odds of normal follow-up sleep (OR = 2.81, 95% CI: 1.64–4.82, *p* < 0.001), whereas no additional linear association was observed at scores of 14 or higher (OR = 0.99, 95% CI: 0.83–1.19, *p* = 0.948). Restricting the analysis to participants with ISI ≥ 14 produced a similar null upper-range association (OR = 1.05, 95% CI: 0.87–1.26, *p* = 0.632) (Supplementary Table S5). No evidence of ISI nonlinearity was observed in the life-satisfaction model (spline comparison *p* = 0.398).

The ISI × life satisfaction interaction was sensitive to functional form. It was statistically significant in the initial linear summary model (*p* = 0.015) and in a joint spline-interaction test (χ^2^ = 9.53, df = 3, *p* = 0.023), but not in the quadratic model (*p* = 0.063), piecewise model (*p* = 0.207), or the analysis restricted to ISI ≥ 14 (*p* = 0.202) (Supplementary Table S10). The interaction was therefore regarded as exploratory and model-dependent.

When baseline sleep quality was omitted from the spline-based indirect-effect analysis, the average indirect association remained statistically significant (estimate = 0.0119, 95% bootstrap CI: 0.0046–0.0207, *p* < 0.001), whereas the average direct association (estimate = −0.0003, 95% bootstrap CI: −0.0532–0.0548, *p* = 0.953) and total local association (estimate = 0.0117, 95% bootstrap CI: −0.0429–0.0664, *p* = 0.721) were not significant (Supplementary Table S8). In component-specific linear analyses, ISI was associated with adequate sleep restfulness at follow-up (OR = 1.20, 95% CI: 1.06–1.36, *p* = 0.004) but not with adequate sleep duration (OR = 1.03, 95% CI: 0.89–1.19, *p* = 0.676) (Supplementary Table S3). Variance inflation factors ranged from 1.05 to 2.40 (Supplementary Table S7).

## 4. Discussion

This three-year longitudinal cohort study found an association between baseline social interaction and normal sleep quality at follow-up after adjustment for baseline sleep quality, anxiety symptoms, depressive symptoms, and other covariates. However, the association was nonlinear: sleep probability increased primarily across the lower ISI range and plateaued near the upper end. ISI was also associated with life satisfaction. A spline-based exploratory analysis showed an indirect association through life satisfaction, whereas evidence of ISI × life satisfaction effect modification varied across parameterizations. In component-specific analyses, ISI was associated with sleep restfulness but not sleep duration.

The nonlinear findings are central to interpretation. The natural cubic spline and piecewise models indicated that the association was concentrated below approximately 14 ISI points. At scores of 14 or higher, each additional point was not associated with further improvement in follow-up sleep probability. Because most participants scored in this upper range, the original linear OR of 1.24 should be interpreted as an average summary across a heterogeneous curve rather than as a constant dose–response relationship. The apparent plateau may reflect saturation of social resources, limited discrimination near the scale ceiling, or both.

Evidence of effect modification by life satisfaction was less robust than suggested by the single linear interaction term. The interaction remained significant in the spline model but was not confirmed in the quadratic, piecewise, or upper-range-restricted analyses. It may therefore be influenced by the relatively small lower-ISI tail and by modeling choices. Subgroup interpretations should remain exploratory, and the results do not show that increasing social interaction would necessarily improve sleep among adults dissatisfied with their current lives.

The spline-based natural-effect analysis identified a small local indirect association through life satisfaction, while the direct and total local associations were not statistically significant. A significant indirect association in the absence of a significant total local association is statistically possible, but it does not establish mediation. Social interaction and life satisfaction were measured concurrently at baseline; their temporal ordering cannot be established, and unmeasured common causes may contribute to both. The result is therefore best described as an exploratory indirect statistical association near the sample mean.

The component-specific findings add measurement detail to the composite outcome. Baseline ISI was associated with adequate sleep restfulness at follow-up but not with sleeping at least 6 h. Social relationships and subjective well-being may be more closely related to perceived restoration, emotional arousal, stress, and the subjective experience of sleep than to sleep duration itself. This interpretation is consistent with evidence that subjective and objective sleep indicators do not always correspond [24]. It also means that the principal composite association should not be interpreted as evidence that social interaction lengthens sleep duration.

The findings are broadly consistent with systematic evidence connecting social relationships, social isolation, and loneliness with sleep and wider health outcomes [8,9,10] and with Japanese research showing that social relationships and physical activity jointly relate to sleep disorders in older adults [11]. Social interaction may be associated with sleep through emotional support, reduced loneliness, stress buffering, belonging, meaningful activity, and more regular daily rhythms [25,26,27,28,29,30,31]. However, most cited studies differ in age, measurement, and cultural context, and the present sample consisted of working-age adults rather than exclusively older adults. The current study, therefore, extends, but does not directly replicate, older-adult findings.

Cross-cultural interpretation is especially important because social participation and subjective well-being are embedded in family structures, community norms, and religious practices. Religious involvement may provide social networks, shared routines, meaning, and psychological resources that are relevant to sleep [12], while qualitative evidence indicates that religious prescriptions and prayer practices may also alter sleep timing and duration [13]. Preliminary findings in Greek older adults have additionally examined sleep quality and general health in relation to religiousness [14]. Culturally specific forms of mutual help, such as Osekkai in rural Japan, may support social contact and community participation during periods of disruption [15]. Multidimensional research in older adults likewise indicates that life satisfaction is shaped by social, psychological, physical, and environmental conditions [16]. The present cohort did not assess religiosity or specific forms of cultural participation, so the observed interaction between ISI and life satisfaction should not be assumed to operate identically in populations with different religious, social, or cultural characteristics.

Anxiety symptoms were added as a covariate because anxiety is closely related to sleep and may be longitudinally intertwined with loneliness and sleep quality in community populations [1,32]. Population-based longitudinal evidence has also shown that insomnia can precede subsequent anxiety and depression [33]. In clinical research on bipolar disorder, commentary has emphasized the importance of assessing insomnia-related sleep disruption alongside cognition and concurrent anxiety [34]. In the present models, anxiety was not independently associated with follow-up sleep after adjustment, and its inclusion did not materially change the principal ISI results. This does not indicate that anxiety is unimportant; the single binary item may have had limited sensitivity, and anxiety may operate through pathways or symptom severity not captured by the available measure.

The timing of baseline data collection also requires consideration. The baseline survey was conducted in August 2020 during the COVID-19 pandemic, when social contact, family interaction, community activities, psychological well-being, and sleep may have differed from pre-pandemic conditions. Among Japanese workers, greater time spent with family was associated with lower loneliness [35], while loneliness and limited interpersonal communication were strongly associated with psychological distress [36]. Rural Japanese research also suggested that culturally embedded mutual-help activities could support social participation during the pandemic [15], and nationwide Japanese data documented differences in nonrestorative sleep during the pandemic period [5]. Consequently, baseline ISI, life satisfaction, psychological symptoms, and sleep responses may partly reflect the specific social and behavioral circumstances of the pandemic, limiting direct generalization to periods without comparable disruption.

Attrition analyses showed that 38.8% of baseline participants were not included in the final complete-case analysis. Included participants were younger (absolute SMD = 0.28), whereas no other examined baseline characteristic differed statistically, including the five dietary indicators, current drinking, depressive symptoms, and the baseline sleep indicators. Selection bias cannot be excluded because age differed between groups, and reasons for loss to follow-up were unavailable, although the remaining standardized differences were small. In addition, the total number of participants invited to the baseline survey was unavailable, preventing calculation of the initial participation rate.

The upper-end ISI concentration also affected precision. Nearly half of the participants scored 17 or 18, while only 70 scored 14 or lower. The spline and piecewise estimates at the lower end were therefore based on a relatively small subgroup and had wider uncertainty. Conversely, the densely represented upper range showed little evidence of an additional linear association. This distribution may also help explain why the unadjusted median comparison was not statistically significant despite an average adjusted linear association.

The results have cautious clinical relevance. Sleep complaints are important across adulthood and may be considered alongside mental health, functional, and neurocognitive information in clinical and community settings; clinical commentary has similarly highlighted concurrent consideration of insomnia-related disruption, cognition, and anxiety [34]. Cross-cultural work on dementia assessment emphasizes that functional measures require cultural validity and adaptation [37]. However, the present study did not measure cognition, dementia, diagnostic status, or predictive accuracy. It therefore cannot support conclusions about neurocognitive diagnosis or claim that the psychosocial indicators identify individuals with a clinical sleep disorder.

The broader relevance of social connection is supported by meta-analytic and conceptual evidence linking social relationships with mortality and wider health outcomes [38,39]. Research on social support, interpersonal need support, psychological resilience, and social connectedness further indicates consistent associations with well-being and life satisfaction across different populations and life stages [40,41,42,43,44,45].

Sleep-specific research has also linked social relationships and social support with sleep quality in community and occupational populations [46,47,48]. Additional evidence suggests interconnected relationships among sleep, quality of life, perceived stress, depressive symptoms, mental health, and life satisfaction [49,50,51].

The interpretation of life satisfaction as a brief subjective well-being indicator is additionally informed by conceptual work distinguishing life satisfaction from emotional well-being and by research supporting the validity of single-item assessment in large population samples [52,53,54].

This study also has several strengths. It used a three-year longitudinal outcome; adjusted for baseline sleep status; included anxiety and depressive symptoms; compared included and non-included participants; separated sleep duration from sleep restfulness; retained four directional sleep transitions; and assessed ISI functional form using spline, quadratic, piecewise, and restricted-range analyses. It also evaluated the robustness of the interaction across parameterizations and estimated local natural associations under a nonlinear outcome model. The multidimensional ISI and guideline-based sleep definition were relevant to the Japanese community context.

### 4.1. Limitations

Several limitations should be acknowledged. First, social interaction and life satisfaction were assessed at the same baseline wave. The temporal ordering required for causal mediation was therefore not established, and the indirect association may reflect reverse directionality or shared unmeasured determinants. The natural effect terminology describes the statistical estimands used and should not be interpreted as proof of a causal mechanism.

Second, sleep duration and sleep restfulness were self-reported. The composite outcome was aligned with Japanese sleep-health guidance, but it did not capture insomnia symptoms, sleep timing, variability, sleep efficiency, sleep disorders, medication use, or objective physiology. The separate analyses further showed that the ISI association was evident for sleep restfulness rather than sleep duration, limiting broad interpretation of the outcome as a uniform construct.

Third, life satisfaction, depressive symptoms, and anxiety symptoms were assessed using brief binary items rather than validated multi-item scales or clinical interviews. These measures reduced respondent burden but could not characterize symptom severity, duration, diagnostic status, or the multidimensional nature of subjective well-being. Measurement error may have attenuated or otherwise altered the observed associations.

Fourth, the study used non-probability sampling and complete-case analysis. The denominator of residents initially invited was unavailable, so the baseline participation rate could not be calculated. Of the 796 baseline respondents, 309 were not included in the final analysis, and an age difference was observed. Selection and healthy-participant biases may therefore limit representativeness.

Fifth, the August 2020 baseline occurred during the COVID-19 pandemic. Social interaction, life satisfaction, lifestyle, and sleep may have been influenced by pandemic-related circumstances, as suggested by Japanese evidence on loneliness, family interaction, community participation, psychological distress, and nonrestorative sleep during this period [5,15,35,36]. The available data could not isolate those effects, and the findings may not fully represent associations under usual community conditions.

Sixth, the study was conducted in one suburban Japanese community. Socioeconomic status, occupation and work schedules, caregiving burden, loneliness, detailed social-network characteristics, religiousness, and other cultural factors were not fully assessed. Residual confounding remains possible, and replication is needed in culturally and socioeconomically diverse populations.

Seventh, the ISI showed pronounced upper-end concentration and a nonlinear association with the sleep outcome. Although spline and piecewise analyses addressed the functional form, only 70 participants scored 14 or lower, so the steep lower-range association was estimated from a relatively small subgroup. The interaction with life satisfaction was also sensitive to ISI parameterization and should not be treated as a stable subgroup effect. The four sleep-transition groups were reported descriptively rather than modeled extensively because the normal-to-poor group was small. These analyses should be considered exploratory.

### 4.2. Implications for Healthcare and Community Health Promotion

The findings suggest that very low social-interaction scores may be more informative for sleep-health research than small differences near the upper end of the ISI scale. The plateau indicates that the results should not be interpreted as a simple dose–response relationship. Life satisfaction may be relevant to this association, but the interaction was model-dependent and should be used to generate hypotheses rather than to define a screening subgroup. The study did not evaluate predictive performance, screening thresholds, or intervention effectiveness.

Community health initiatives may consider interpersonal relationships, meaningful participation, subjective well-being, and sleep-restfulness complaints alongside conventional sleep behaviors. Such consideration should complement, not replace, clinical assessment or evidence-based sleep care. The present findings do not establish that programs designed to increase social interaction or life satisfaction will improve sleep.

Future studies should use repeated measurements of social interaction and life satisfaction across multiple waves, validated multi-item psychological and sleep instruments, objective sleep measures, and diverse cultural settings. Prospective prediction studies and intervention trials will be needed before these psychosocial indicators can be recommended for screening, risk classification, or prevention strategies.

## 5. Conclusions

Baseline social interaction was associated with normal sleep quality at three-year follow-up among community-dwelling adults in Japan, but the association was nonlinear and concentrated in the lower ISI range, with a plateau at scores of approximately 14 or higher. Life satisfaction showed a small exploratory indirect association near the mean ISI score, while evidence of effect modification was sensitive to model specification. The association was more evident for sleep restfulness than for sleep duration. Because social interaction and life satisfaction were measured concurrently, these findings do not establish causal mediation. Replication with broader ISI distributions, repeated psychosocial assessments, and validated or objective sleep measures is needed.

## Figures and Tables

**Table 1:** Baseline characteristics of the analytic sample.

Characteristic	Total (*n* = 487)
Baseline age, years	43.84 ± 9.10
BMI, kg/m^2^	22.80 ± 3.69
BMI category	
<18.5 kg/m^2^	37 (7.6)
18.5–24.9 kg/m^2^	337 (69.2)
≥25 kg/m^2^	113 (23.2)
Index of Social Interaction score	17 [15–17]
Sex (female)	288 (59.1)
Normal sleep quality at baseline	221 (45.4)
Life satisfaction	367 (75.4)
Breakfast consumption	434 (89.1)
Snack intake	212 (43.5)
Salt intake control	343 (70.4)
Attention to nutritional balance	387 (79.5)
Avoidance of high-fat foods	264 (54.2)
Exercise	219 (45.0)
Current smoking	73 (15.0)
Current drinking	231 (47.4)
Hospitalization/prolonged treatment	103 (21.1)
Depressive symptoms	43 (8.8)
Anxiety symptoms	64 (13.1)

Note. Values are presented as mean ± SD, median [interquartile range], or *n* (%).

**Table 2:** Baseline characteristics according to follow-up sleep quality.

Characteristic	Poor Sleep Quality (*n* = 210)	Normal Sleep Quality (*n* = 277)	*p*-Value
Baseline age, years	44.02 ± 8.29	43.70 ± 9.68	0.692
BMI, kg/m^2^	23.16 ± 3.95	22.53 ± 3.45	0.069
Index of Social Interaction score	16 [15–17]	17 [16–17]	0.176
Sex (female)	125 (59.5)	163 (58.8)	0.880
Normal sleep quality at baseline	42 (20.0)	179 (64.6)	<0.001
Life satisfaction	120 (57.1)	247 (89.2)	<0.001
Breakfast consumption	183 (87.1)	251 (90.6)	0.223
Snack intake	96 (45.7)	116 (41.9)	0.398
Salt intake control	151 (71.9)	192 (69.3)	0.535
Attention to nutritional balance	164 (78.1)	223 (80.5)	0.514
Avoidance of high-fat foods	111 (52.9)	153 (55.2)	0.602
Exercise	78 (37.1)	141 (50.9)	0.003
Current smoking	39 (18.6)	34 (12.3)	0.054
Current drinking	96 (45.7)	135 (48.7)	0.508
Hospitalization/prolonged treatment	46 (21.9)	57 (20.6)	0.722
Depressive symptoms	22 (10.5)	21 (7.6)	0.265
Anxiety symptoms	34 (16.2)	30 (10.8)	0.083

Note. Values are mean ± SD, median [interquartile range], or *n* (%). Comparisons are descriptive, and *p*-values are unadjusted.

**Table 3:** Initial linear summary associations and exploratory interaction results.

Association or Effect	Model	OR (95% CI)	*p*-Value
ISI and normal follow-up sleep quality	Linear total-association model	1.24 (1.09–1.42)	<0.001
ISI and life satisfaction	Linear mediator model	1.32 (1.16–1.51)	<0.001
Life satisfaction and normal follow-up sleep quality	Linear interaction model; evaluated at mean ISI	4.78 (2.71–8.44)	<0.001
ISI and normal follow-up sleep among participants not satisfied with their current lives	Conditional linear association	1.50 (1.14–1.97)	0.003
ISI and normal follow-up sleep among participants satisfied with their current lives	Conditional linear association	1.03 (0.87–1.23)	0.698
Linear ISI × life satisfaction	Linear interaction model	0.69 (0.50–0.95)	0.022

Note. These models use a linear ISI parameterization. The per-point ISI OR is an average association across the observed score range. ISI was mean-centered at 16.11 points in the interaction model, and the OR for life satisfaction is evaluated at the mean ISI score. All models adjusted for age, sex, BMI, baseline sleep quality, dietary variables, exercise, current smoking, current drinking, hospitalization/prolonged treatment, depressive symptoms, and anxiety symptoms. The linear interaction yielded a likelihood-ratio χ^2^ = 5.93, *p* = 0.015, but its robustness under alternative ISI parameterizations is reported in Supplementary Table S10.

**Table 4:** Spline-based exploratory natural direct and indirect associations for a local 1-point ISI contrast.

Effect	Estimate	Boot SE	95% Bootstrap CI	*p*-Value
Natural indirect association with outcome evaluated at mean ISI	0.0068	0.0038	0.0002–0.0150	0.045
Natural indirect association with outcome evaluated at mean ISI + 1	0.0086	0.0035	0.0028–0.0164	0.005
Average natural indirect association	0.0077	0.0034	0.0019–0.0153	0.009
Natural direct association under mediator distribution at mean ISI	0.0106	0.0256	−0.0417–0.0619	0.711
Natural direct association under mediator distribution at mean ISI + 1	0.0124	0.0258	−0.0386–0.0651	0.661
Average natural direct association	0.0115	0.0257	−0.0403–0.0637	0.689
Total local association	0.0192	0.0258	−0.0336–0.0717	0.464

Note. Estimates are on the risk-difference scale for an increase in ISI from 16.11 to 17.11 points. The mediator model used a linear ISI term, while the outcome model used a natural cubic spline for ISI and spline × life satisfaction interaction terms. Both models adjusted for the same covariates, including anxiety symptoms. Confidence intervals were estimated using 3000 bootstrap resamples. These estimates describe exploratory local statistical associations and should not be interpreted as causal mediation.

## Data Availability

The data that support the findings of this study are not publicly available because of ethical and privacy restrictions related to the community-based cohort survey. Data may be made available from the corresponding author upon reasonable request and with permission from the relevant ethics committee and local municipal authority. The statistical analysis code is available from the corresponding author upon reasonable request.

## Supplementary Materials

The following supporting information can be downloaded at: https://www.mdpi.com/article/10.3390/healthcare14152311/s1: Supplementary Table S1, Baseline comparison between included and non-included participants; Supplementary Table S2, Distribution and overlap of sleep-duration and sleep-restfulness criteria; Supplementary Table S3, Component-specific adjusted logistic regression analyses; Supplementary Table S4, Four directional sleep-quality transitions from baseline to follow-up; Supplementary Table S5, Alternative ISI parameterizations and assessment of nonlinearity for follow-up normal sleep quality; Supplementary Table S6, Initial linear summary adjusted logistic-regression models including anxiety symptoms; Supplementary Table S7, Multicollinearity assessment for the full interaction outcome model; Supplementary Table S8, Spline-based indirect-effect sensitivity analysis without adjustment for baseline sleep quality; Supplementary Table S9, Assessment and coding of study variables; Supplementary Table S10, Robustness of ISI functional form and the ISI × life satisfaction interaction; Supplementary Figure S1, Covariate-adjusted probability of normal sleep quality at follow-up according to ISI score from the natural cubic spline model. Observed proportions are shown for comparison; the dashed vertical line marks the piecewise-model knot at ISI = 14. Scores below 10 were omitted from the plot because only two participants had such scores; Supplementary Table S11, Index of Social Interaction (ISI).

## Abbreviations

The following abbreviations are used in this manuscript:

BMI	Body mass index
CI	Confidence interval
ISI	Index of Social Interaction
OR	Odds ratio
STROBE	Strengthening the Reporting of Observational Studies in Epidemiology
VIF	Variance inflation factor
