# Supplementary material for: Social Interaction, Life Satisfaction, and Follow-Up Sleep Quality in Community-Dwelling Adults: A Three-Year Longitudinal Cohort Study in Japan
Source: Healthcare (Basel). 2026 Jul 31;14(15):2311. doi: 10.3390/healthcare14152311 (PMC13465278; doi:10.3390/healthcare14152311)
Supplement: Supplementary file 1 [file healthcare-14-02311-s001.zip › healthcare-4445402-supplementary.pdf]

healthcare

MDPI

Supplementary Materials

Supplementary Table S1. Baseline comparison between included and non-included participants.

Characteristic	Included (n = 487)	Not included (n = 309)	p value	Absolute SMD
Baseline age, years	43.84 ± 9.10	46.21 ± 7.93	<0.001	0.28
BMI, kg/m²	22.80 ± 3.69	22.73 ± 3.54	0.774	0.02
Index of Social Interaction score	17 [15–17]	17 [15–17]	0.714	0.00
Sex (female)	288 (59.1)	165 (53.4)	0.111	0.12
Life satisfaction	367 (75.4)	235 (76.1)	0.825	0.02
Breakfast every day	434 (89.1)	274 (88.7)	0.846	0.01
Snack intake	212 (43.5)	144 (46.6)	0.396	0.06
Salt intake control	343 (70.4)	224 (72.5)	0.531	0.05
Attention to nutritional balance	387 (79.5)	251 (81.2)	0.543	0.04
Avoidance of high-fat foods	264 (54.2)	179 (57.9)	0.303	0.07
Exercise	219 (45.0)	156 (50.5)	0.129	0.11
Current smoking	73 (15.0)	46 (14.9)	0.968	0.00
Current drinking	231 (47.4)	152 (49.2)	0.629	0.04
Hospitalization/prolonged treatment	103 (21.1)	76 (24.6)	0.256	0.08
Depressive symptoms	43 (8.8)	18 (5.8)	0.120	0.12
Anxiety symptoms	64 (13.1)	50 (16.2)	0.233	0.09
Adequate sleep duration at baseline	400 (82.1)	246 (79.6)	0.375	0.06
Adequate sleep restfulness at baseline	224 (46.0)	145 (46.9)	0.798	0.02
Normal sleep quality at baseline	221 (45.4)	143 (46.3)	0.804	0.02

Note. Values are mean ± SD, median [interquartile range], or n (%). The not-included group comprised participants lost to follow-up or excluded for other reasons. Continuous variables were compared using Welch t tests, the ISI score using the Mann–Whitney U test, and categorical variables using Pearson chi-square tests. Comparisons are descriptive and p values are unadjusted. SMD = standardized mean difference. All five dietary indicators were available in the updated baseline file and were included in this comparison.

Supplementary Table S2. Distribution and overlap of sleep-duration and sleep-restfulness criteria.

Time point	Sleep category	n	%
Baseline (2020)	Both duration and restfulness adequate	221	45.4
Baseline (2020)	Short duration only	3	0.6
Baseline (2020)	Insufficient restfulness only	179	36.8
Baseline (2020)	Both short duration and insufficient restfulness	84	17.2
Follow-up (2023)	Both duration and restfulness adequate	277	56.9
Follow-up (2023)	Short duration only	5	1.0
Follow-up (2023)	Insufficient restfulness only	125	25.7
Follow-up (2023)	Both short duration and insufficient restfulness	80	16.4

Supplementary Table S3. Component-specific adjusted logistic regression analyses.

Follow-up outcome	Effect	OR (95% CI)	p value	Interaction LR χ^2	Interaction LR p value
Adequate sleep duration at follow-up	ISI, total association model	1.03 (0.89–1.19)	0.676	1.17	0.280
Adequate sleep duration at follow-up	ISI among participants not satisfied with current life	0.89 (0.72–1.10)	0.296	1.17	0.280
Adequate sleep duration at follow-up	ISI among participants satisfied with current life	1.06 (0.85–1.32)	0.621	1.17	0.280
Adequate sleep duration at follow-up	Life satisfaction at mean ISI	4.12 (2.30–7.38)	<0.001	1.17	0.280
Adequate sleep duration at follow-up	ISI × life satisfaction	1.18 (0.87–1.61)	0.282	1.17	0.280
Adequate sleep restfulness at follow-up	ISI, total association model	1.20 (1.06–1.36)	0.004	3.38	0.066
Adequate sleep restfulness at follow-up	ISI among participants not satisfied with current life	1.33 (1.05–1.68)	0.017	3.38	0.066
Adequate sleep restfulness at follow-up	ISI among participants satisfied with current life	1.02 (0.86–1.21)	0.799	3.38	0.066
Adequate sleep restfulness at follow-up	Life satisfaction at mean ISI	4.50 (2.58–7.84)	<0.001	3.38	0.066
Adequate sleep restfulness at follow-up	ISI × life satisfaction	0.77 (0.58–1.03)	0.074	3.38	0.066

Note. These component-specific models used a linear ISI term and adjusted for the corresponding baseline sleep component and all other covariates, including depressive and anxiety symptoms. ISI was mean-centered in models containing the interaction. The principal nonlinear analyses concerned the composite follow-up sleep outcome.

Supplementary Table S4. Four directional sleep-quality transitions from baseline to follow-up.

Sleep-quality transition	n	%	Baseline ISI score	Life satisfaction, n (%)	Anxiety symptoms, n (%)
Normal → Normal	179	36.8	17.0 [15.0–17.0]	162 (90.5)	11 (6.1)
Normal → Poor	42	8.6	16.0 [15.0–17.0]	29 (69.0)	6 (14.3)
Poor → Normal	98	20.1	17.0 [16.0–17.0]	85 (86.7)	19 (19.4)
Poor → Poor	168	34.5	16.5 [15.0–17.0]	91 (54.2)	28 (16.7)

Note. The former favorable/unfavorable trajectory variable was removed because it was effectively equivalent to follow-up sleep status. ISI is presented as median [interquartile range].

Supplementary Table S5. Alternative ISI parameterizations and assessment of nonlinearity for follow-up normal sleep quality.

Model	ISI effective df	AIC	Comparison	LR χ^2	df	p value
Covariate-only model	0	582.64	Reference	—	—	—
Linear ISI	1	572.70	vs. covariate-only	11.95	1	<0.001
Quadratic ISI	2	563.76	vs. linear	10.94	1	<0.001
Natural cubic spline	3	557.01	vs. linear	19.69	2	<0.001
Piecewise linear; knot = 14	2	561.36	vs. linear	13.34	1	<0.001

Note. All models adjusted for baseline age, sex, BMI, baseline normal sleep quality, dietary indicators, exercise, current smoking, current drinking, hospitalization/prolonged treatment, depressive symptoms, and anxiety symptoms. The natural cubic spline used three effective degrees of freedom. In the piecewise model, the OR per 1-point increase was 2.81 (95% CI: 1.64–4.82; $p < 0.001$) below 14 and 0.99 (95% CI: 0.83–1.19; $p = 0.948$) at 14 or higher. In the analysis restricted to ISI ≥14 (n = 455), the OR was 1.05 (95% CI: 0.87–1.26; $p = 0.632$).

Observed ISI score distribution

ISI score	n	%
6	1	0.2
9	1	0.2
10	4	0.8
11	6	1.2
12	9	1.8
13	11	2.3
14	38	7.8
15	64	13.1
16	108	22.2
17	154	31.6
18	91	18.7

Supplementary Table S6. Initial linear summary adjusted logistic-regression models including anxiety symptoms.

Variable	Model 1 OR (95% CI)	p value	Model 2 OR (95% CI)	p value	Model 3 OR (95% CI)	p value
Index of Social Interaction score	1.24 (1.09–1.42)	<0.001	1.32 (1.16–1.51)	<0.001	1.50 (1.14–1.97)	0.003
Life satisfaction	—	—	—	—	4.78 (2.71–8.44)	<0.001
ISI × life satisfaction	—	—	—	—	0.69 (0.50–0.95)	0.022
Baseline age, years	1.00 (0.97–1.02)	0.746	0.99 (0.96–1.02)	0.383	1.00 (0.98–1.03)	0.873
Female	0.93 (0.56–1.53)	0.768	0.74 (0.42–1.32)	0.313	1.01 (0.60–1.71)	0.971
BMI	0.95 (0.89–1.00)	0.069	0.89 (0.84–0.95)	<0.001	0.97 (0.91–1.03)	0.298
Normal sleep quality at baseline	7.51 (4.83–11.70)	<0.001	3.27 (1.96–5.46)	<0.001	6.76 (4.25–10.76)	<0.001
Breakfast consumption	1.23 (0.61–2.47)	0.565	3.41 (1.67–6.99)	<0.001	0.83 (0.40–1.75)	0.627
Snack intake	0.92 (0.59–1.44)	0.706	0.81 (0.49–1.35)	0.415	0.99 (0.62–1.57)	0.954
Salt intake control	0.61 (0.33–1.16)	0.131	0.97 (0.48–1.95)	0.927	0.60 (0.31–1.17)	0.132
Attention to nutritional balance	1.17 (0.59–2.36)	0.651	1.22 (0.57–2.61)	0.608	1.08 (0.51–2.29)	0.830
Avoidance of high-fat foods	0.98 (0.59–1.64)	0.950	1.20 (0.68–2.14)	0.530	0.96 (0.56–1.63)	0.876
Exercise	1.62 (1.05–2.50)	0.029	1.26 (0.77–2.07)	0.353	1.58 (1.00–2.49)	0.048
Current smoking	0.59 (0.32–1.08)	0.085	0.80 (0.41–1.56)	0.514	0.59 (0.31–1.10)	0.099
Current drinking	1.11 (0.71–1.73)	0.647	1.00 (0.60–1.67)	0.989	1.09 (0.69–1.73)	0.710
Hospitalization/prolonged treatment	1.12 (0.66–1.90)	0.685	0.79 (0.44–1.41)	0.429	1.10 (0.63–1.95)	0.733
Depressive symptoms	0.89 (0.43–1.85)	0.760	0.16 (0.08–0.34)	<0.001	1.67 (0.73–3.79)	0.224
Anxiety symptoms	0.84 (0.46–1.52)	0.566	1.07 (0.55–2.09)	0.849	0.78 (0.41–1.45)	0.428

Note. Model 1 outcome: follow-up normal sleep quality. Model 2 outcome: life satisfaction. Model 3 outcome: follow-up normal sleep quality and includes a linear ISI × life satisfaction interaction. These models use a linear ISI term, so the per-point OR represents an average association across the observed ISI range. In Model 3, ISI is mean-centered; its coefficient is conditional on not being satisfied with current life, and the coefficient for life satisfaction is evaluated at the mean ISI score. Alternative functional forms are reported in Supplementary Tables S5 and S10.

Supplementary Table S7. Multicollinearity assessment for the full interaction outcome model.

Variable	VIF
Mean-centered ISI score	2.40
ISI × life satisfaction	2.31
Attention to nutritional balance	1.88
Salt intake control	1.87
Avoidance of high-fat foods	1.50
Female	1.46
Life satisfaction	1.27
BMI	1.17
Snack intake	1.16
Current drinking	1.16
Current smoking	1.16
Hospitalization/prolonged treatment	1.14
Breakfast consumption	1.14
Baseline age	1.14
Normal sleep quality at baseline	1.11
Depressive symptoms	1.10
Exercise	1.10
Anxiety symptoms	1.05

Note. The maximum VIF was 2.40. Mean-centering was used before constructing the interaction term. No VIF exceeded 2.5.

Supplementary Table S8. Spline-based indirect-effect sensitivity analysis without adjustment for baseline sleep quality.

Effect	Estimate	Boot SE	95% bootstrap CI	p value
Natural indirect association with outcome evaluated at mean ISI	0.0110	0.0046	0.0030–0.0206	0.007
Natural indirect association with outcome evaluated at mean ISI + 1	0.0129	0.0042	0.0057–0.0221	<0.001
Average natural indirect association	0.0119	0.0042	0.0046–0.0207	<0.001
Natural direct association under mediator distribution at mean ISI	−0.0012	0.0279	−0.0543–0.0534	0.915
Natural direct association under mediator distribution at mean ISI + 1	0.0007	0.0279	−0.0523–0.0563	0.977
Average natural direct association	−0.0003	0.0279	−0.0532–0.0548	0.953
Total local association	0.0117	0.0283	−0.0429–0.0664	0.721

Note. The mediator model used a linear ISI term. The outcome model used a natural cubic spline for ISI and spline × life satisfaction interaction terms. All covariates except baseline sleep quality were included. Estimates are on the risk-difference scale for a local increase from ISI = 16.11 to 17.11. Confidence intervals were estimated using 3,000 bootstrap resamples. These are exploratory statistical associations rather than causal mediation effects.

Supplementary Table S9. Assessment and coding of study variables.

Variable	Assessment item or measurement	Original response options	Coding used in the analysis
Baseline age	Age at the August 2020 baseline assessment.	Recorded in completed years.	Continuous variable.
Sex	Self-reported sex.	Male; Female.	0 = male; 1 = female.
BMI	Calculated from height and weight measured at a health examination centre operated by the local government.	Height recorded in metres and weight recorded in kilograms.	BMI = weight (kg)/height (m)^2; treated as a continuous variable.
Index of Social Interaction score	The 18-item Index of Social Interaction; the full item list is provided in Supplementary Table S11.	Each item was answered according to the original binary response format.	Each item was coded 0 or 1 and summed; continuous total score ranging from 0 to 18.
Life satisfaction	"Are you satisfied with your current life?"	Yes; No.	1 = yes; 0 = no.
Sleep duration	"Approximately how many hours and minutes do you sleep per day?"	Recorded in hours and minutes.	1 = at least 6 hours; 0 = less than 6 hours.
Sleep restfulness	"Do you think you obtain sufficient rest through sleep?"	Yes; No.	1 = yes; 0 = no.
Normal sleep quality	Derived from sleep duration and sleep restfulness.	Not applicable.	1 = sleep duration of at least 6 hours and adequate sleep restfulness; 0 = either criterion not met.
Breakfast consumption	"Do you eat breakfast every day?"	Yes; No.	1 = yes; 0 = no.
Snack intake	"Do you eat snacks between meals?"	Yes; No.	1 = yes; 0 = no.
Salt intake control	"Do you try to limit your salt intake?"	Yes; No.	1 = yes; 0 = no.
Attention to nutritional balance	"Do you pay attention to maintaining a nutritionally balanced diet?"	Yes; No.	1 = yes; 0 = no.
Avoidance of high-fat foods	"Do you limit foods that are high in fat?"	Yes; No.	1 = yes; 0 = no.
Exercise	"Do you engage in any exercise?"	Almost every day; 3–4 times per week; 1–2 times per week; Almost never.	1 = almost every day, 3–4 times per week, or 1–2 times per week; 0 = almost never.

Variable	Assessment item or measurement	Original response options	Coding used in the analysis
Current smoking	"Do you currently smoke cigarettes?"	Almost every day; Sometimes; Former smoker; Never smoked.	1 = almost every day or sometimes; 0 = former smoker or never smoked.
Current drinking	"Do you drink alcohol?"	Almost every day; Sometimes; Never.	1 = almost every day or sometimes; 0 = never.
Hospitalization/prolonged treatment	"During the past year, have you been hospitalized or received continuous treatment for an illness for at least two weeks?"	Yes; No.	1 = yes; 0 = no.
Depressive symptoms	"During the past year, have you felt down or depressed?"	Yes; No.	1 = yes; 0 = no.
Anxiety symptoms	"During the past year, have you experienced feelings of anxiety or worry?"	Yes; No.	1 = yes; 0 = no.

Note. The questionnaire was administered in Japanese, and English translations of the assessment items are presented here. Depressive and anxiety symptoms were measured using brief single-item self-reported indicators and should not be interpreted as clinical diagnoses.

Supplementary Table S10. Robustness of ISI functional form and the ISI × life satisfaction interaction.

Analysis	Parameterization or contrast	Estimate/statistic	df	p value	Interpretation
Nonlinear shape	Piecewise slope below ISI 14	OR = 2.81 (95% CI: 1.64–4.82)	—	<0.001	Positive association in lower range
Nonlinear shape	Piecewise slope at ISI 14 or higher	OR = 0.99 (95% CI: 0.83–1.19)	—	0.948	Plateau in upper range
Upper-range sensitivity	Linear ISI among participants with ISI ≥14	OR = 1.05 (95% CI: 0.87–1.26)	—	0.632	No upper-range linear association
Interaction robustness	Linear ISI × life satisfaction	LR χ^2 = 5.93	1	0.015	Statistically significant
Interaction robustness	Joint spline × life satisfaction terms	LR χ^2 = 9.53	3	0.023	Statistically significant
Interaction robustness	Joint quadratic ISI × life satisfaction terms	LR χ^2 = 5.54	2	0.063	Not statistically significant
Interaction robustness	Joint piecewise ISI × life satisfaction terms	LR χ^2 = 3.15	2	0.207	Not statistically significant
Interaction robustness	Linear interaction restricted to ISI ≥14	LR χ^2 = 1.63	1	0.202	Not statistically significant

Note. Interaction likelihood-ratio tests compare models with and without the corresponding interaction terms while retaining the same main-effect parameterization and covariates. The inconsistent interaction results indicate that effect modification by life satisfaction is exploratory and sensitive to ISI functional form.

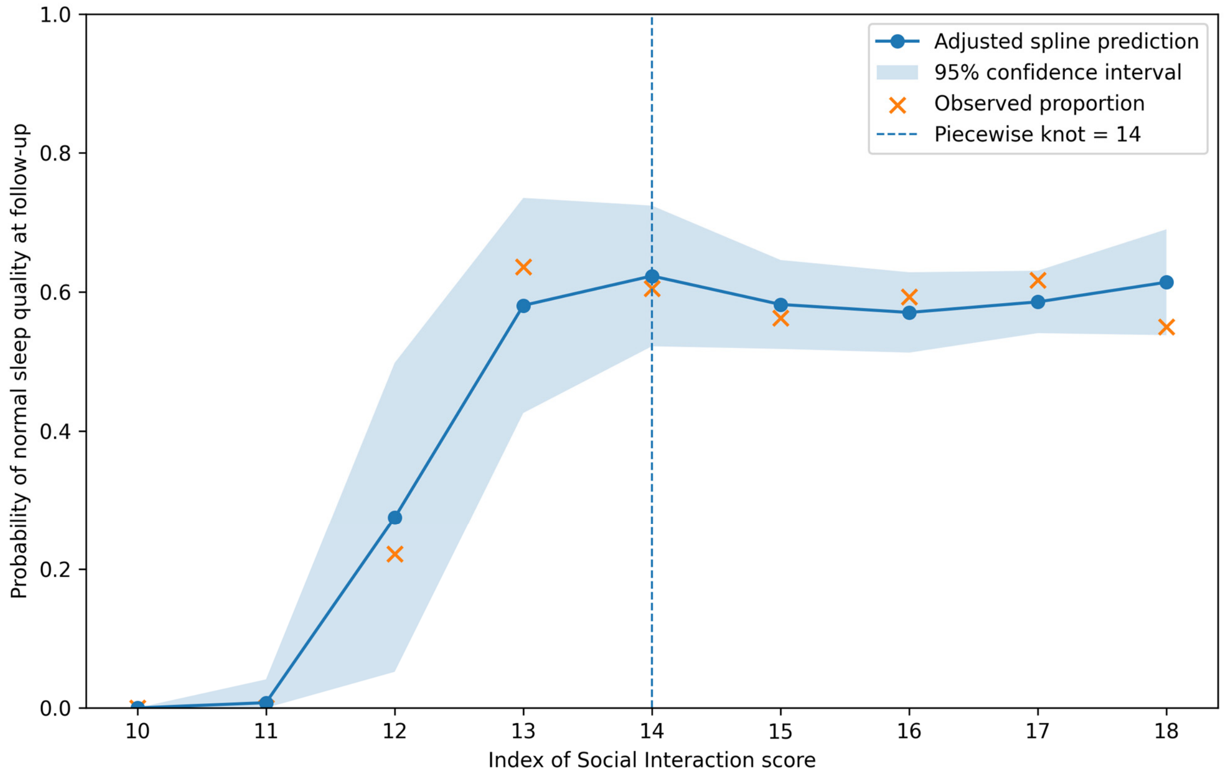

Supplementary Figure S1. Covariate-adjusted probability of normal sleep quality at follow-up according to ISI score from the natural cubic spline model. Observed proportions are shown for comparison; the dashed vertical line marks the piecewise-model knot at ISI = 14. Scores below 10 were omitted from the plot because only two participants had such scores.

Supplementary Table S11. Index of Social Interaction (ISI)

Items	Questions	Options
Motivation to live (own way)	“Do you have motivation to live an active lifestyle?”	(1) Yes (2) No
Taking an active approach	“Do you take an active approach toward your life?”	(1) Yes (2) No
Being inspired to lead a healthy lifestyle	“Are you motivated to live a healthy life?”	(1) Yes (2) No
Maintaining a routine way of life	“Do you have a regular or routine lifestyle?”	(1) Yes (2) No
Reading newspapers	“Are you a reader of newspaper?”	(1) Yes (2) No
Reading books	“Do you read books or magazines regularly?”	(1) Yes (2) No
Try to use new equipment	“Do you try to use new equipment?”	(1) Yes (2) No
Having a hobby	“Do you have any hobbies?”	(1) Yes (2) No
Feeling of importance	“Do you have feeling of importance in the society?”	(1) Yes (2) No
Family communication	“Do you often communicate with your family members?”	(1) Yes (2) No
Interaction with nonfamily persons	“Do you interact with nonfamily persons regularly?”	(1) Yes (2) No
Engagement with social groups	“Do you have chance to participate in social groups?”	(1) Yes (2) No
Watching television	“Do you watch television?”	(1) Yes (2) No
Involvement in community events	“Do you have chance to participate in your neighborhood affairs?”	(1) Yes (2) No
Frequency of friendships	“How often do you make friends?”	(1) Yes (2) No
Having an active role in society	“Do you have an active role in the society or social affairs?”	(1) Yes (2) No
Having counsel	“Do you have someone to counsel with in difficult situations?”	(1) Yes (2) No
Having someone to give support in emergency	“Do you have someone to support you in emergency?”	(1) Yes (2) No
